# Re: Evaluation of erectile function after anastomotic vs substitution urethroplasty for bulbar stricture. Omar RG et al., Arab J Urol2020 [Epub ahead of print]. DOI:10.1080/2090598x.2020.1805965

**DOI:** 10.1080/2090598X.2020.1832402

**Published:** 2020-10-28

**Authors:** Michael S. Floyd Jr, Ahmad M. Omar, Andrew D. Baird, Paul CB Anderson

**Affiliations:** Consultant Urological Surgeons, Department of Reconstructive Urology, St Helens and Knowsley, NHS Trust, Whiston Hospital, Liverpool, UK; Consultant Urological Surgeon, Department of Reconstructive Urology, Aintree University Hospital, Liverpool, UK; Consultant Urological Surgeon, Department of Genitourethral Reconstruction, Russell’s Hall Hospital, Dudley, West Midlands, UK

Dear Editor,

We read with interest the recent paper by Omar *et al*. [1] regarding the evaluation of erectile function (EF) following bulbar urethroplasty. A prospective non-randomised study of 34 patients with stricture who underwent either anastomotic or substitution urethroplasty is presented with specific emphasis on the effects of the procedure on EF. A total of 21 patients underwent anastomotic urethroplasty and 13 underwent substitution urethroplasty with either buccal or penile skin graft. All patients were assessed pre- and postoperatively with the International Index of Erectile Function (IIEF)-15 questionnaire. All patients with pre-existing erectile dysfunction (ED) were excluded, but the authors do not elaborate on the diabetic status, smoking history or the use of relevant medications known to cause ED in the study population preoperatively.

The results concluded that: (a) there was a difference between mean and postoperative IIEF scores in the anastomotic group at both the 3- and 6-month follow-up, but (b) no difference was noted in the substitution urethroplasty group; furthermore, when the mean change in IIEF score was compared in both groups over the study period no difference was noticed; and overall (c) the study demonstrated that any type of bulbar urethroplasty had no statistically significant impact on EF [1].

The study again highlights one of the challenges facing urethral surgeons: measuring sexual function following urethroplasty [2]. The IIEF and its short-form variant although commonly used are not specific for urethral disease [3]. The Brief Male Sexual Function Inventory (BMFSI) is often used to assess male sexual function, but again it lacks specificity for urethral disease [4]. The validated Men’s Health Sexual Questionnaire (MHSQ) is not specific for urethral disease, but has been used to assess ejaculatory function following buccal graft for staged penile urethroplasty [5,6].

The authors reference a paper by Barbagli *et al*. [7] (using a non-validated patient-reported outcome measure to specifically examine the effects of urethroplasty on sexual function), stating that 153 patients with bulbar stricture who underwent anastomotic urethroplasty reported no ED at 5 years; however, that same study demonstrated that 23% of patients’ studied reported ejaculatory dysfunction and this is relevant.

The authors are to be commended for performing this study examining the impact of urethroplasty on EF specifically, but should acknowledge that the sample size is small and only a single questionnaire was used to assess EF. Urkmez *et al*. [8] in 2019 published a series of 60 patients who underwent substitution or anastomotic urethroplasty and reported no impact on EF or orgasmic function, but acknowledged the lack of validated questionnaires available to assess sexual function after urethroplasty.

Ideally, successful outcome assessment following urethral surgery should objectively evaluate voiding and all sexual outcomes, in addition to quality-of-life improvements with a validated, reproducible questionnaire.

Breyer *et al*. [9] have developed a 32-item Urethral Stricture Symptoms and Impact measure (USSIM), which assesses both voiding and sexual outcomes after urethral reconstructive surgery.
